# Supplementary material for: Nonmonotonic particle-size-dependence of magnetoelectric coupling in strained nanosized particles of BiFeO3
Source: Sci Rep. 2018 Feb 27;8:3728. doi: 10.1038/s41598-018-21803-1 (PMC5829220; doi:10.1038/s41598-018-21803-1)
Supplement: Supplementary file 1 — Supplementary Information [file 41598_2018_21803_MOESM1_ESM.pdf]

Supplementary Information

Nonmonotonic particle-size-dependence of magnetoelectric coupling in strained nano-sized particles of $BiFeO_3$

Sudipta Goswami[1], Dipten Bhattacharya[2], Chandan K. Ghosh[3], Barnali Ghosh[4], S.D. Kaushik[5], V. Siruguri[5], and P.S.R. Krishna[6]

[1]Department of Solid State Physics, Indian Association for the Cultivation of Science, Kolkata 700032, India, [2]Nanostructured Materials Division, CSIR-Central Glass and Ceramic Research Institute, Kolkata 700032, India, [3]School of Materials Science and Nanotechnology, Jadavpur University, Kolkata 700032, India, [4]Department of Materials Science, S.N. Bose National Centre for Basic Sciences, Kolkata 70098, India, [5]UGC-DAE Consortium for Scientific Research, Bhabha Atomic Research Centre, Mumbai 400085, India, [6]Solid State Physics Division, Bhabha Atomic Research Centre, Mumbai 400085, India

We provide here the representative TEM/HRTEM images and powder x-ray and neutron diffraction data for bulk and nanoscale $BiFeO_3$. We also show the representative results obtained from Rietveld refinement of the diffraction data for different samples. The fit statistics and other structural details are mentioned for a few selected cases. The plots of T_N, lattice strain and lattice volume as a function of particle size are shown. The theoretically determined basis functions corresponding to the displacement modes τ_1, τ_2, and τ_3 for the Fe(6a) and O(18b) ions in R3c structure within the framework of isostructural transition at T_N have been mentioned. Finally, the displacements of the Fe and O ions corresponding to the modes τ_1 and τ_2 are also shown using those theoretically determined basis functions.

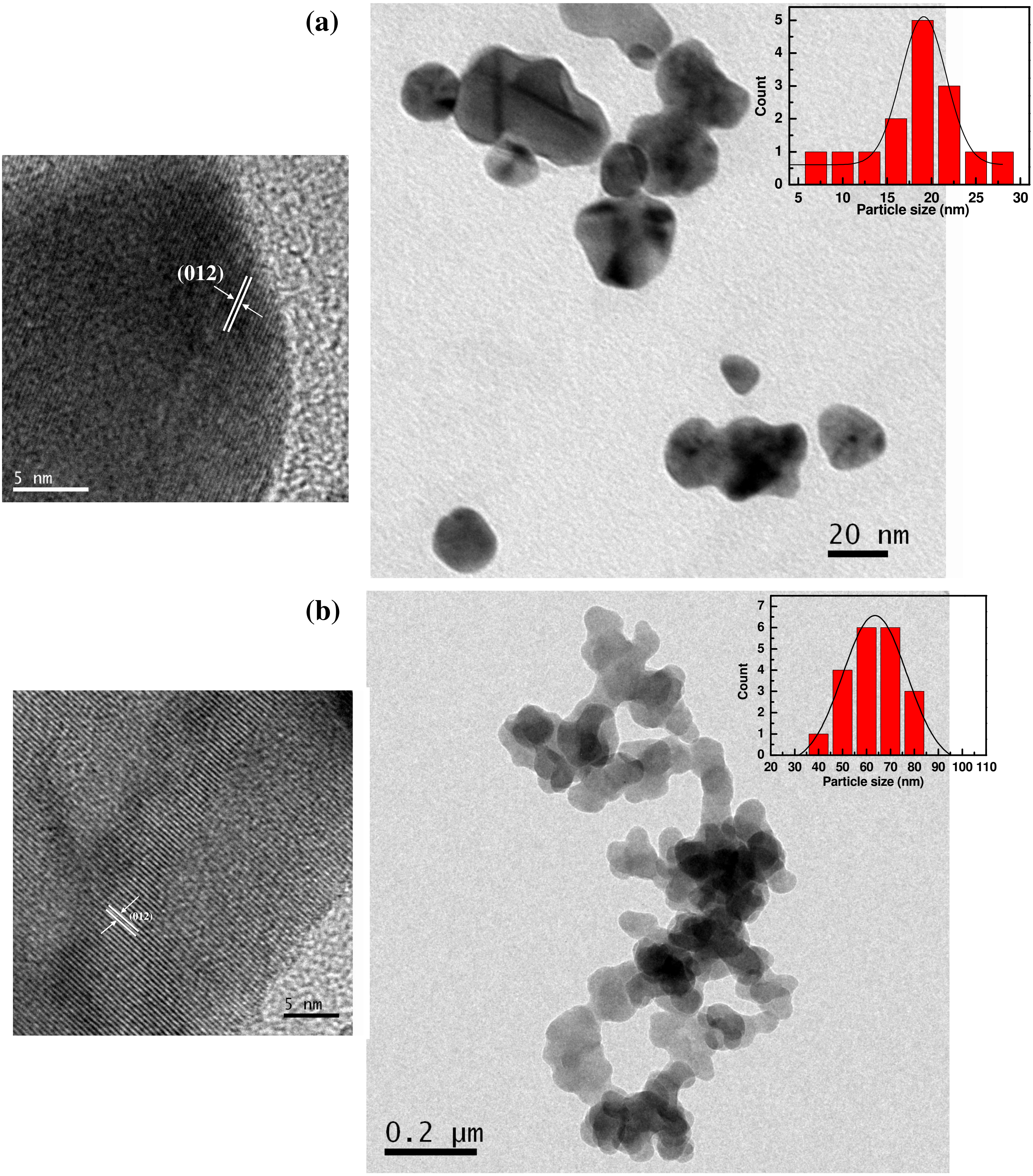

Fig. S1. Representative TEM and HRTEM images of (a) finer (~20 nm) and (b) coarser (~60 nm) particles of $BiFeO_3$.

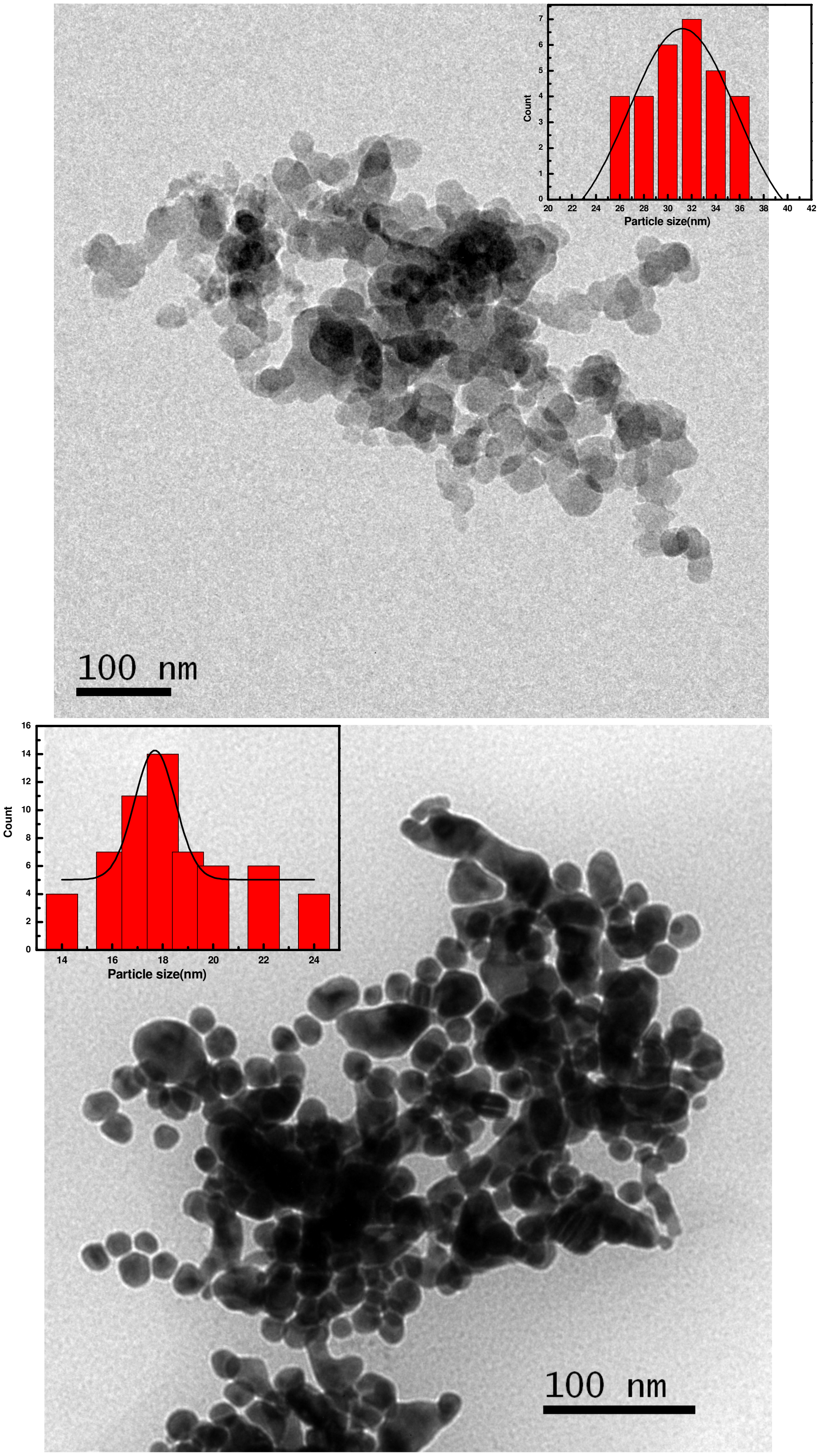

Fig. S2. Additional TEM images of nanoparticles of $BiFeO_3$ of different sizes (~20 nm, ~30 nm).

The lattice strain has been calculated from the HRTEM data shown above which yields the ‘d’ value of the crystallographic plane (lattice fringe). The corresponding bulk ‘d’ value was noted from the JCPDS card (No. 86-1518). For example, for the (012) plane the ‘d’ values obtained from HRTEM data in different samples appear to be ~3.958 and ~3.939 Å. The bulk ‘d’ value is ~3.9628 Å. The lattice strain was calculated from $(d_{exp}-d_{jcpds})/d_{exp}$, where d_{exp} is the experimentally observed value. The lattice strain turns out to be ~0.12% and ~0.6%. Finer particles exhibit higher strain. These results are consistent with what has been found from the analysis of the x-ray/neutron diffraction data.

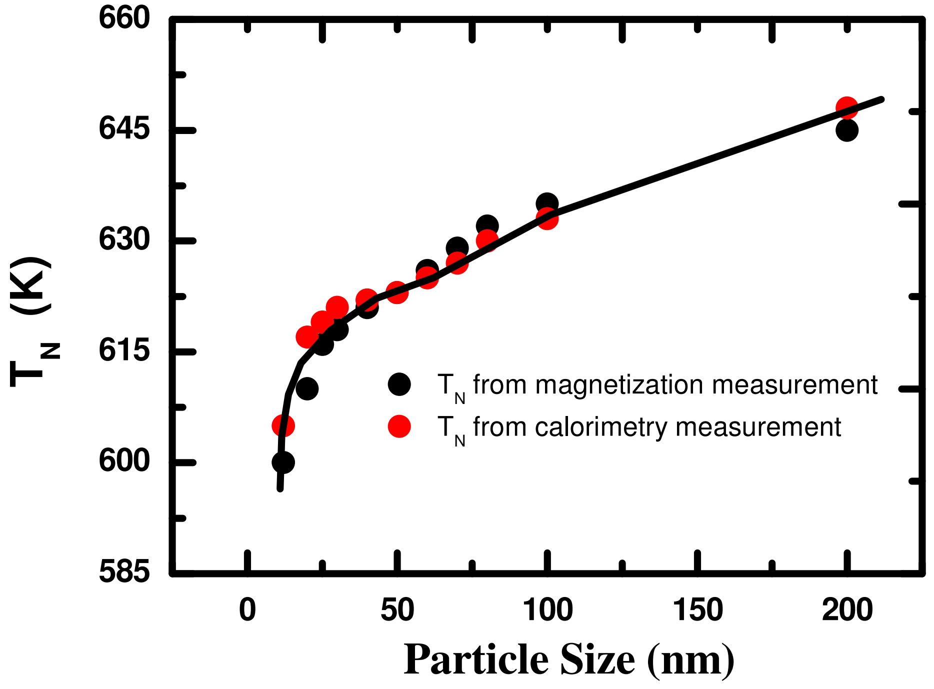

Fig. S3. Particle size dependence of T_N.

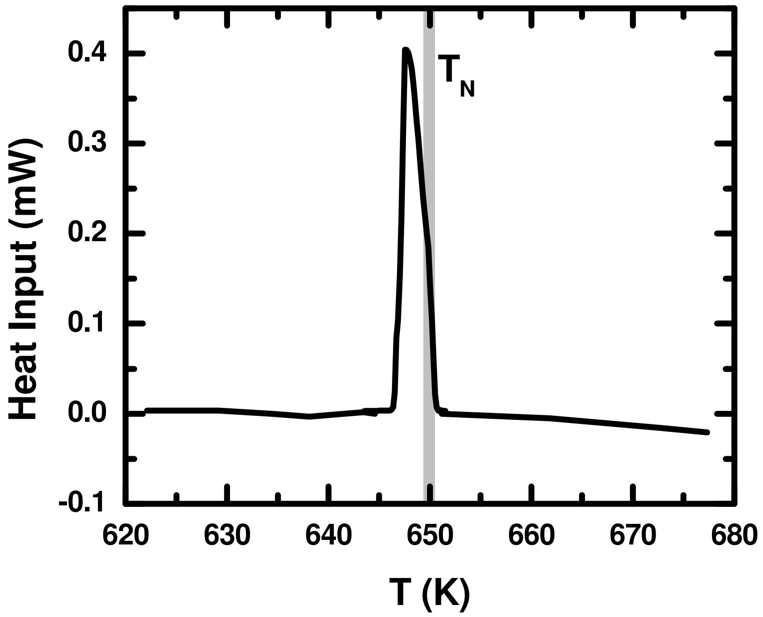

Fig. S4. The calorimetric (power compensated) trace for the bulk $BiFeO_3$ around T_N.

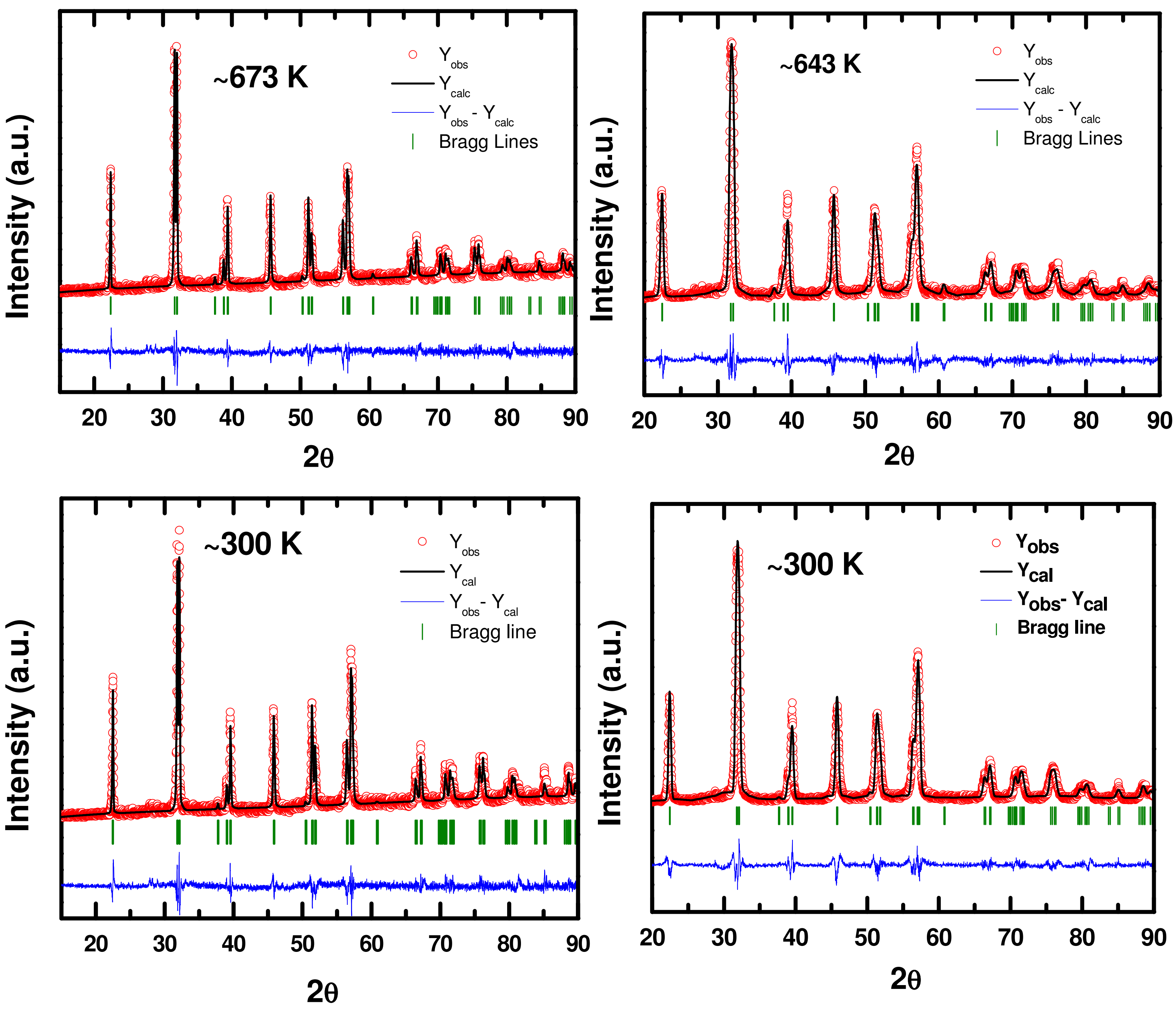

Fig. S5. Laboratory x-ray diffraction patterns for bulk (~200 nm) and nanoscale (~30 nm) $BiFeO_3$ and room and higher temperatures.

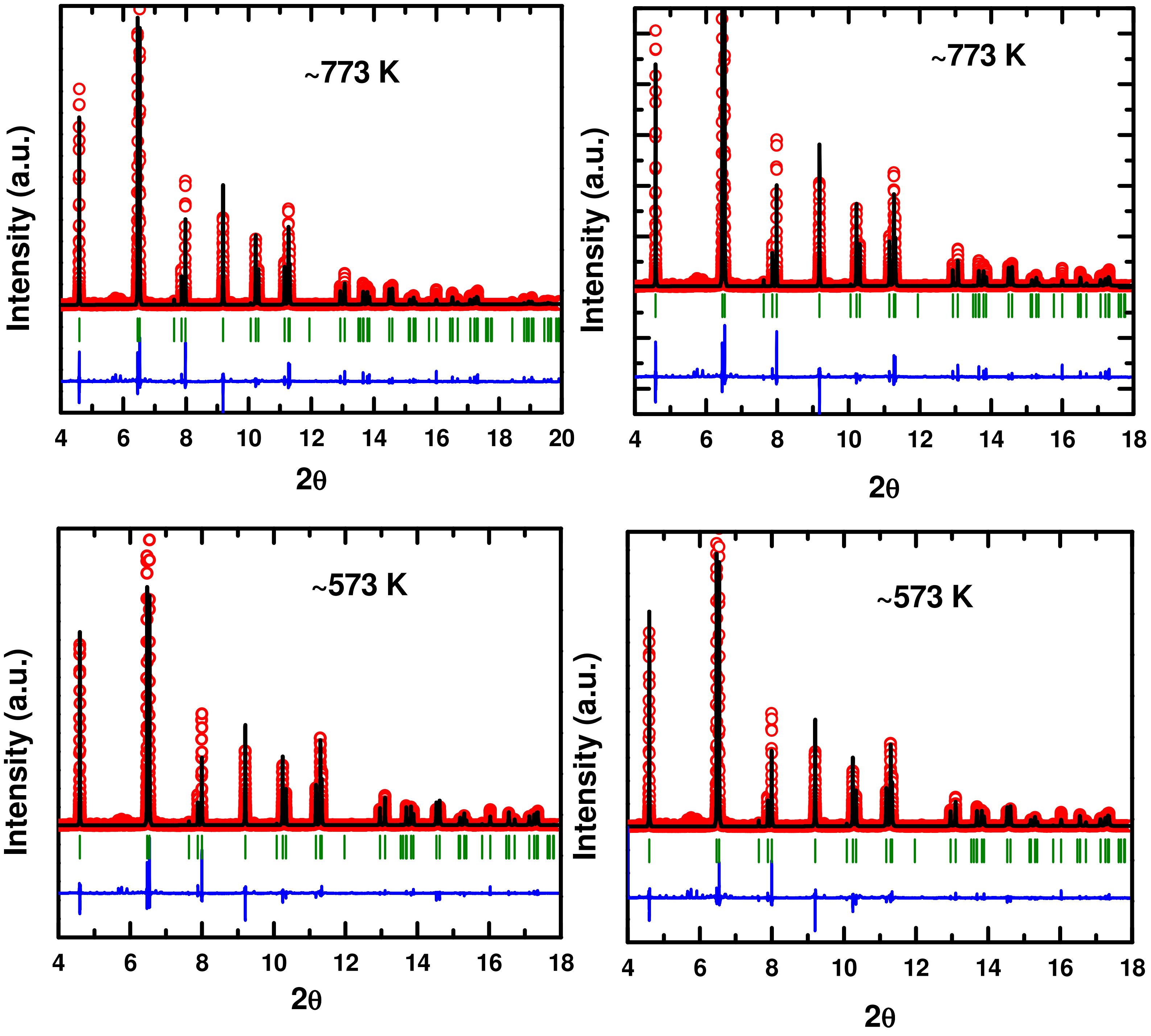

Fig. S6. Synchrotron x-ray diffraction data (ESRF) for (a) ~100 nm and (b) ~60 nm $BiFeO_3$.

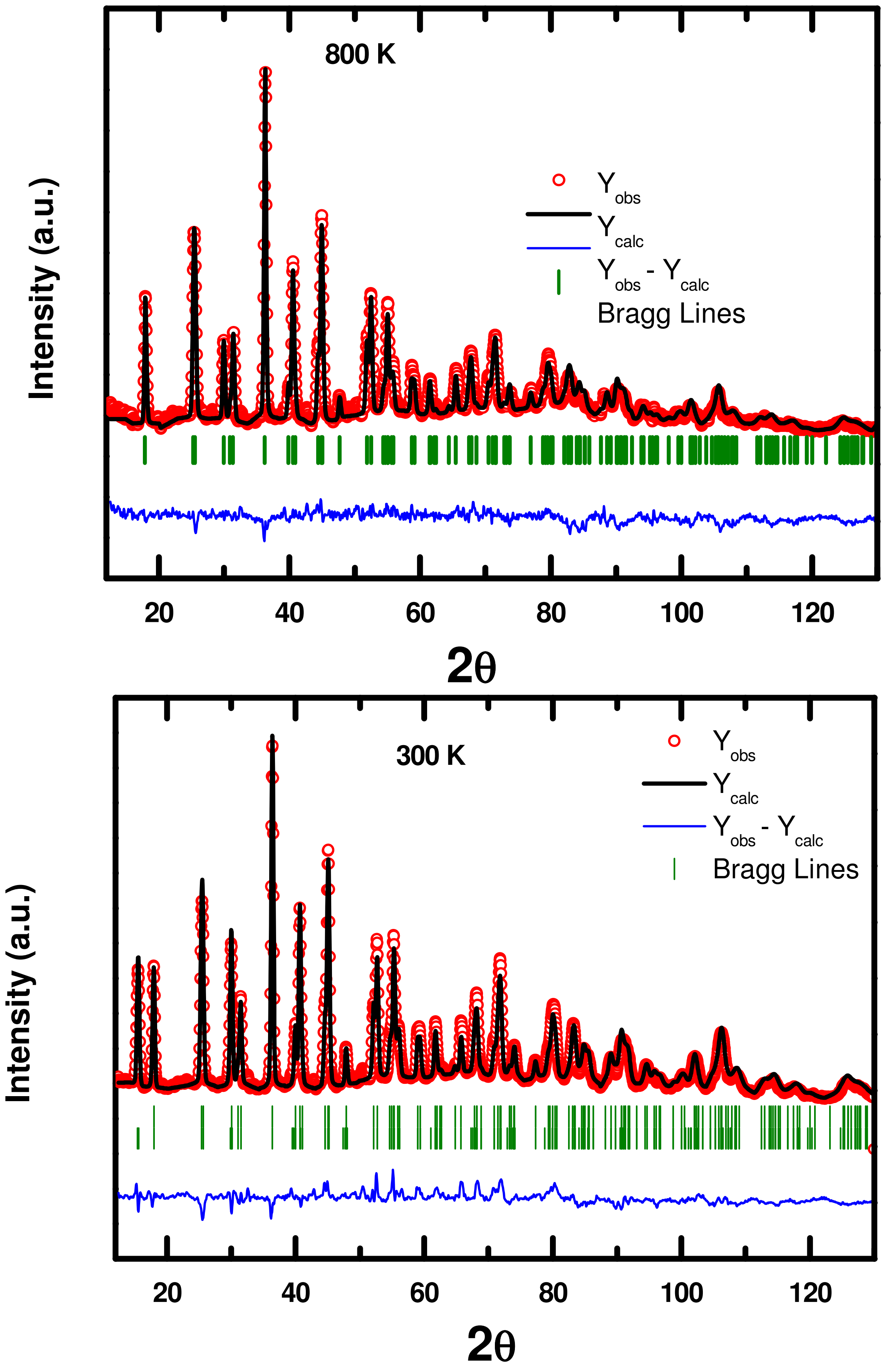

Fig. S7. Neutron diffraction data for bulk $BiFeO_3$ (~200 nm) at below and above T_N.

Table S-I: Fit statistics of Rietveld refinement of X-Ray and Neutron diffraction patterns and other structural details for a few representative samples at different temperature

(A) Bulk $BiFeO_3$

**Temp (K)**	R_p	R_{wp}	χ^2	**Bond Length (Å)**	***Bond Angle (°)***
298	9.09	11.6	2.42	Bi-O= 2.5633<br>Bi-O= 2.5436<br>Fe-O= 1.6708	O-Fe-O= 90.4234<br>O-Bi-O= 80.7905
373	8.94	11.3	2.28	Bi-O= 2.5825<br>Bi-O= 2.5178<br>Fe-O= 1.7306	O-Fe-O = 89.0777<br>O-Bi-O= 78.7859
473	8.70	11.1	2.18	Bi-O= 2.5718<br>Bi-O= 2.5221<br>Fe-O= 1.7256	O-Fe-O = 90.9089<br>O-Bi-O= 79.1945
523	8.42	10.9	2.09	Bi-O= 2.5644<br>Bi-O= 2.5191<br>Fe-O= 1.7235	O-Fe-O = 90.7933<br>O-Bi-O= 79.5288
573	8.57	11.0	2.14	Bi-O= 2.5653<br>Bi-O= 2.5183<br>Fe-O= 1.7368	O-Fe-O = 91.3475<br>O-Bi-O= 78.7267
628	8.47	10.9	2.09	Bi-O= 2.5727<br>Bi-O= 2.5045<br>Fe-O= 1.7262	O-Fe-O = 91.0553<br>O-Bi-O= 78.9864
633	8.20	10.5	1.94	Bi-O= 2.5704<br>Bi-O= 2.5179<br>Fe-O= 1.7222	O-Fe-O = 91.2343<br>O-Bi-O= 79.2974
638	8.31	10.7	2.04	Bi-O= 2.5728<br>Bi-O= 2.5424<br>Fe-O= 1.7174	O-Fe-O = 89.8429<br>O-Bi-O= 79.4573
643	8.17	10.6	2.02	Bi-O= 2.5803<br>Bi-O= 2.5392<br>Fe-O= 1.715	O-Fe-O = 89.52<br>O-Bi-O= 78.7524
648	8.27	10.6	2.05	Bi-O= 2.6073<br>Bi-O= 2.5362<br>Fe-O= 1.709	O-Fe-O = 89.3012<br>O-Bi-O= 78.6944
653	8.01	10.3	1.96	Bi-O= 2.5913<br>Bi-O= 2.5344<br>Fe-O= 1.7041	O-Fe-O = 89.0654<br>O-Bi-O= 79.3364
658	8.29	10.7	2.11	Bi-O= 2.5904<br>Bi-O= 2.5336<br>Fe-O= 1.6973	O-Fe-O = 89.5032<br>O-Bi-O= 79.4962
663	8.07	10.4	1.93	Bi-O= 2.5728<br>Bi-O= 2.5258<br>Fe-O= 1.696	O-Fe-O = 91.2359<br>O-Bi-O= 79.6419
673	8.25	10.5	1.96	Bi-O= 2.5705<br>Bi-O= 2.5203<br>Fe-O= 1.71	O-Fe-O = 91.794<br>O-Bi-O= 79.7944

(B) 100nm $BiFeO_3$

**Temp (K)**	R_p	R_{wp}	χ^2	**Bond Length (Å)**	***Bond Angle (°)***
298	7.00	9.56	3.06	Bi-O= 2.42<br>Fe-O= 1.7569	O-Fe-O = 83.4689<br>O-Bi-O=
498	8.17	10.7	2.56	Bi-O= 2.4299<br>Fe-O= 1.7091	O-Fe-O = 84.7458<br>O-Bi-O=

518	8.16	10.7	2.61	Bi-O= 2.4429<br>Fe-O= 1.7602	O-Fe-O = 84.8927<br>O-Bi-O=
538	8.09	10.4	2.48	Bi-O= 2.5978<br>Fe-O= 1.7529	O-Fe-O = 84.8334<br>O-Bi-O=
558	8.23	10.6	2.67	Bi-O= 2.4524<br>Fe-O= 1.7798	O-Fe-O = 84.9272<br>O-Bi-O=
578	8.56	11.2	3.00	Bi-O= 2.4405<br>Fe-O= 1.775	O-Fe-O = 84.8053<br>O-Bi-O=
598	8.42	11.1	2.89	Bi-O= 2.4257<br>Fe-O= 1.7327	O-Fe-O = 85.1507<br>O-Bi-O=
618	8.39	11.2	3.06	Bi-O= 2.4272<br>Fe-O= 1.7302	O-Fe-O = 85.3668<br>O-Bi-O=
648	8.93	12.0	3.54	Bi-O= 2.4235<br>Fe-O= 1.7466	O-Fe-O = 86.07<br>O-Bi-O=

(C) 60nm $BiFeO_3$

**Temp (K)**	R_p	R_{wp}	χ^2	**Bond Length (Å)**	***Bond Angle (°)***
298	5.5	7.72	2.68	Bi-O= 2.4714<br>Fe-O= 1.7508	O-Fe-O = 104.4399<br>O-Bi-O= 74.3918
498	5.66	8.75	4.61	Bi-O= 2.0637<br>Fe-O= 1.855	O-Fe-O = 99.0283<br>O-Bi-O=
528	5.49	8.58	4.44	Bi-O= 2.5382<br>Fe-O= 1.7331	O-Fe-O = 96.2654<br>O-Bi-O= 75.6094
538	5.46	8.45	4.32	Bi-O= 2.6622<br>Fe-O= 1.6593	O-Fe-O = 88.65<br>O-Bi-O= 75.9553
548	5.50	8.39	4.23	Bi-O= 2.5875<br>Fe-O= 1.2921	O-Fe-O = 101.3655<br>O-Bi-O= 90.7164
558	5.61	8.46	4.25	Bi-O= 2.5856<br>Fe-O= 1.6559	O-Fe-O = 83.3434<br>O-Bi-O=
568	5.5	8.36	4.19	Bi-O= 2.5796<br>Fe-O= 1.6752	O-Fe-O = 81.8428<br>O-Bi-O=
573	5.52	8.35	4.16	Bi-O= 2.574<br>Fe-O= 1.6815	O-Fe-O = 82.0154<br>O-Bi-O=

(D) 30nm $BiFeO_3$

**Temp(K)**	R_p	R_{wp}	χ^2	**Bond Length (Å)**	***Bond Angle (°)***
298	19.2	23.6	3.22	Bi-O= 2.5561<br>Bi-O=2.5261<br>Fe-O=2.21<br>Fe-O=1.7952	O-Fe-O =91.6135<br>O-Bi-O=75.4442
613	18.1	22.4	2.81	Bi-O= 2.5772<br>Bi-O= 2.4948<br>Fe-O= 2.1966<br>Fe-O= 1.8109	O-Fe-O = 93.7247<br>O-Bi-O= 74.3799
623	18.3	22.7	2.75	Bi-O= 2.5703<br>Bi-O= 2.4994<br>Fe-O= 2.2164<br>Fe-O=1.7947	O-Fe-O = 94.1299<br>O-Bi-O= 74.8237
627	18.3	23.2	2.72	Bi-O= 2.5852<br>Bi-O= 2.4744<br>Fe-O= 2.2155<br>Fe-O=1.7999	O-Fe-O = 88.9202<br>O-Bi-O= 74.7011
629	18.7	24.3	2.82	Bi-O= 2.5928<br>Bi-O= 2.461<br>Fe-O= 2.21<br>Fe-O=1.7953	O-Fe-O = 90.0239<br>O-Bi-O= 74.6933
633	19.5	25.7	3.02	Bi-O= 2.6084<br>Bi-O= 2.4357	O-Fe-O = 96.9559<br>O-Bi-O= 74.4954

				Fe-O= 2.2094<br>Fe-O=1.8022	
638	20.1	26.8	3.20	Bi-O= 2.6225<br>Bi-O= 2.4279<br>Fe-O= 2.1919<br>Fe-O=1.8165	O-Fe-O = 96.9657<br>O-Bi-O= 73.9053
643	20.3	27.0	3.24	Bi-O=2.6131<br>Bi-O=2.4553<br>Fe-O=2.191<br>Fe-O=1.8168	O-Fe-O =95.8089<br>O-Bi-O=73.8318

Bulk $BiFeO_3$ (Neutron Diffraction)

**Temp (K)**	R_p	R_{wp}	χ^2	**Bond Length (Å)**	***Bond Angle (°)***
300	2.02	5.16	6.52	Bi-O= 2.5265<br>Bi-O= 2.3033<br>Fe-O= 2.0972<br>Fe-O= 1.9781	Fe-O-Fe= 89.7302<br>O-Bi-O= 72.6560
340	2.01	5.49	7.44	Bi-O= 2.5273<br>Bi-O= 2.3051<br>Fe-O= 2.0970<br>Fe-O= 1.9786	Fe-O-Fe= 88.2857<br>O-Bi-O= 72.6178
360	2.01	5.36	7.09	Bi-O= 2.5407<br>Bi-O= 2.2930<br>Fe-O= 2.0967<br>Fe-O= 1.9793	Fe-O-Fe= 89.7964<br>O-Bi-O= 72.5454
385	2.03	6.07	8.92	Bi-O= 2.5435<br>Bi-O= 2.2958<br>Fe-O= 2.0906<br>Fe-O= 1.9841	Fe-O-Fe= 89.8779<br>O-Bi-O= 72.3714
410	2.03	5.75	7.99	Bi-O= 2.5420<br>Bi-O= 2.3019<br>Fe-O= 2.0934<br>Fe-O= 1.9828	Fe-O-Fe= 89.8614<br>O-Bi-O= 72.1930
435	2.01	5.49	7.45	Bi-O= 2.5473<br>Bi-O= 2.3014<br>Fe-O= 2.0945<br>Fe-O= 1.9822	Fe-O-Fe= 89.8634<br>O-Bi-O= 72.0730
460	2.05	5.83	8.06	Bi-O= 2.5363<br>Bi-O= 2.3191<br>Fe-O= 2.0990<br>Fe-O= 1.9791	Fe-O-Fe= 89.7991<br>O-Bi-O= 72.0565
475	2.08	5.04	5.87	Bi-O= 2.5299<br>Bi-O= 2.3178<br>Fe-O= 2.1049<br>Fe-O= 1.9766	Fe-O-Fe= 89.7186<br>O-Bi-O= 72.3335
490	2.07	5.67	7.51	Bi-O= 2.5420<br>Bi-O= 2.3163<br>Fe-O= 2.0968<br>Fe-O= 1.9816	Fe-O-Fe= 89.8367<br>O-Bi-O= 71.9865
505	2.01	4.98	6.14	Bi-O= 2.5478<br>Bi-O= 2.3080<br>Fe-O= 2.0926<br>Fe-O= 1.9851	Fe-O-Fe= 89.8921<br>O-Bi-O= 72.1033
520	2.08	4.95	5.68	Bi-O= 2.5447<br>Bi-O= 2.3150<br>Fe-O= 2.1010<br>Fe-O= 1.9793	Fe-O-Fe= 89.8088<br>O-Bi-O= 71.9833
535	2.07	5.07	6.00	Bi-O= 2.5480<br>Bi-O= 2.3110	Fe-O-Fe= 89.7955<br>O-Bi-O= 72.2111

				Fe-O= 2.1022<br>Fe-O= 1.9774	
550	2.04	5.18	6.45	Bi-O= 2.5486<br>Bi-O= 2.3106<br>Fe-O= 2.0932<br>Fe-O= 1.9877	Fe-O-Fe= 89.9224<br>O-Bi-O= 71.7469
565	2.10	4.93	5.50	Bi-O= 2.5479<br>Bi-O= 2.3083<br>Fe-O= 2.0950<br>Fe-O= 1.9871	Fe-O-Fe= 89.9038<br>O-Bi-O= 71.8844
580	2.05	4.84	5.55	Bi-O= 2.5528<br>Bi-O= 2.3082<br>Fe-O= 2.0987<br>Fe-O= 1.9829	Fe-O-Fe= 88.3526<br>O-Bi-O= 71.9457
595	2.11	5.16	5.98	Bi-O= 2.5506<br>Bi-O= 2.3115<br>Fe-O= 2.0932<br>Fe-O= 1.9884	Fe-O-Fe= 88.3895<br>O-Bi-O= 71.8130
610	2.12	4.97	5.52	Bi-O= 2.5499<br>Bi-O= 2.3131<br>Fe-O= 2.1031<br>Fe-O= 1.9803	Fe-O-Fe= 88.3894<br>O-Bi-O= 72.1789
625	2.03	4.93	5.91	Bi-O= 2.5538<br>Bi-O= 2.3143<br>Fe-O= 2.0907<br>Fe-O= 1.9913	Fe-O-Fe= 88.4214<br>O-Bi-O= 71.6371
640	2.08	5.05	5.90	Bi-O= 2.5551<br>Bi-O= 2.3192<br>Fe-O= 2.0998<br>Fe-O= 1.9824	Fe-O-Fe= 88.4469<br>O-Bi-O= 71.8310
680	2.10	5.14	5.98	Bi-O= 2.5540<br>Bi-O= 2.3203<br>Fe-O= 2.0999<br>Fe-O= 1.9848	Fe-O-Fe= 88.4328<br>O-Bi-O= 71.8364
720	2.18	4.86	4.95	Bi-O= 2.5612<br>Bi-O= 2.3152<br>Fe-O= 2.0920<br>Fe-O= 1.9903	Fe-O-Fe= 88.5091<br>O-Bi-O= 71.8384
760	2.11	4.89	5.34	Bi-O= 2.5608<br>Bi-O= 2.3215<br>Fe-O= 2.0947<br>Fe-O= 1.9897	Fe-O-Fe= 88.4628<br>O-Bi-O= 71.5284
800	2.10	5.14	6.01	Bi-O= 2.5665<br>Bi-O= 2.3174<br>Fe-O= 2.0936<br>Fe-O= 1.9890	Fe-O-Fe= 88.4288<br>O-Bi-O= 71.2213

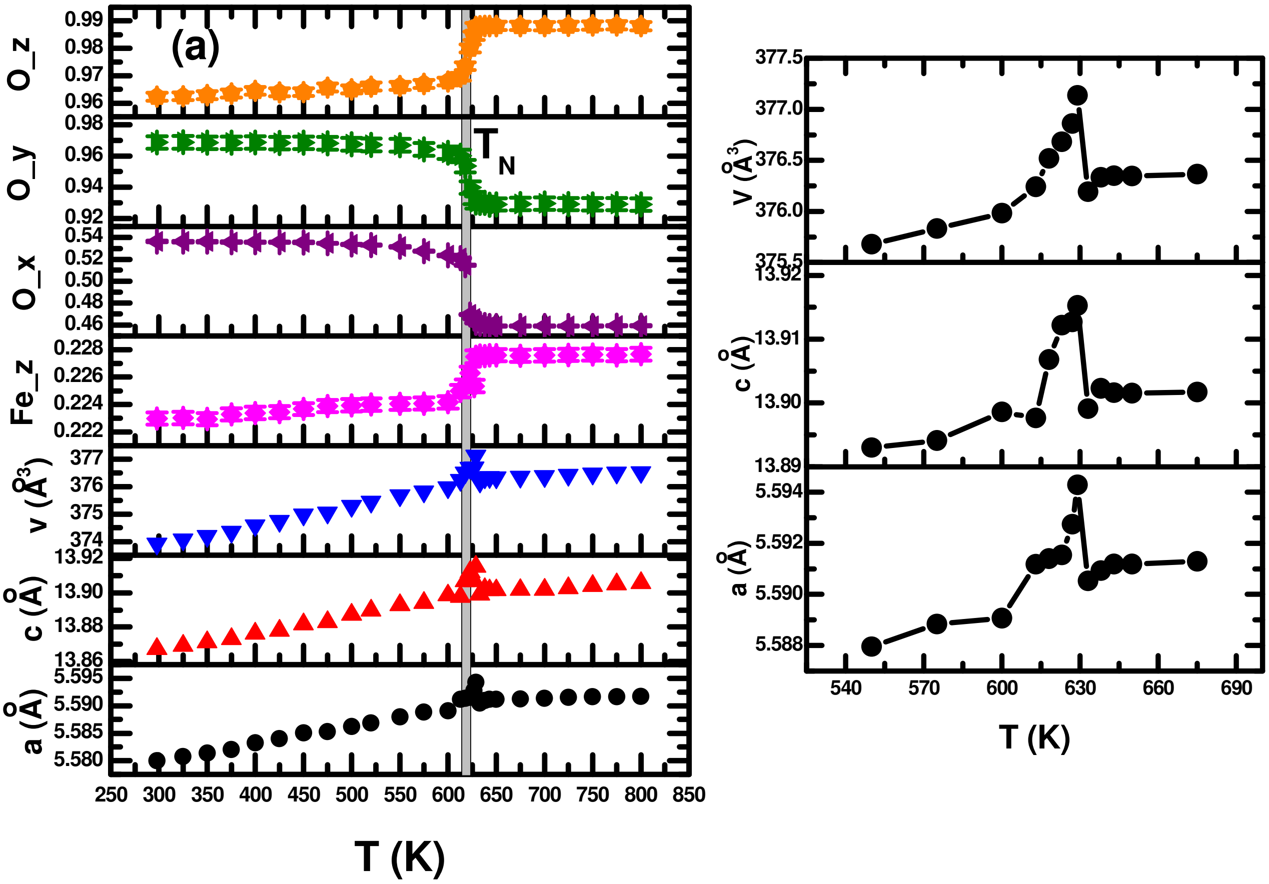
(a)
O_z
O_y
O_x
Fe_z
v (Å³)
c (Å)
a (Å)
T_N
T (K)

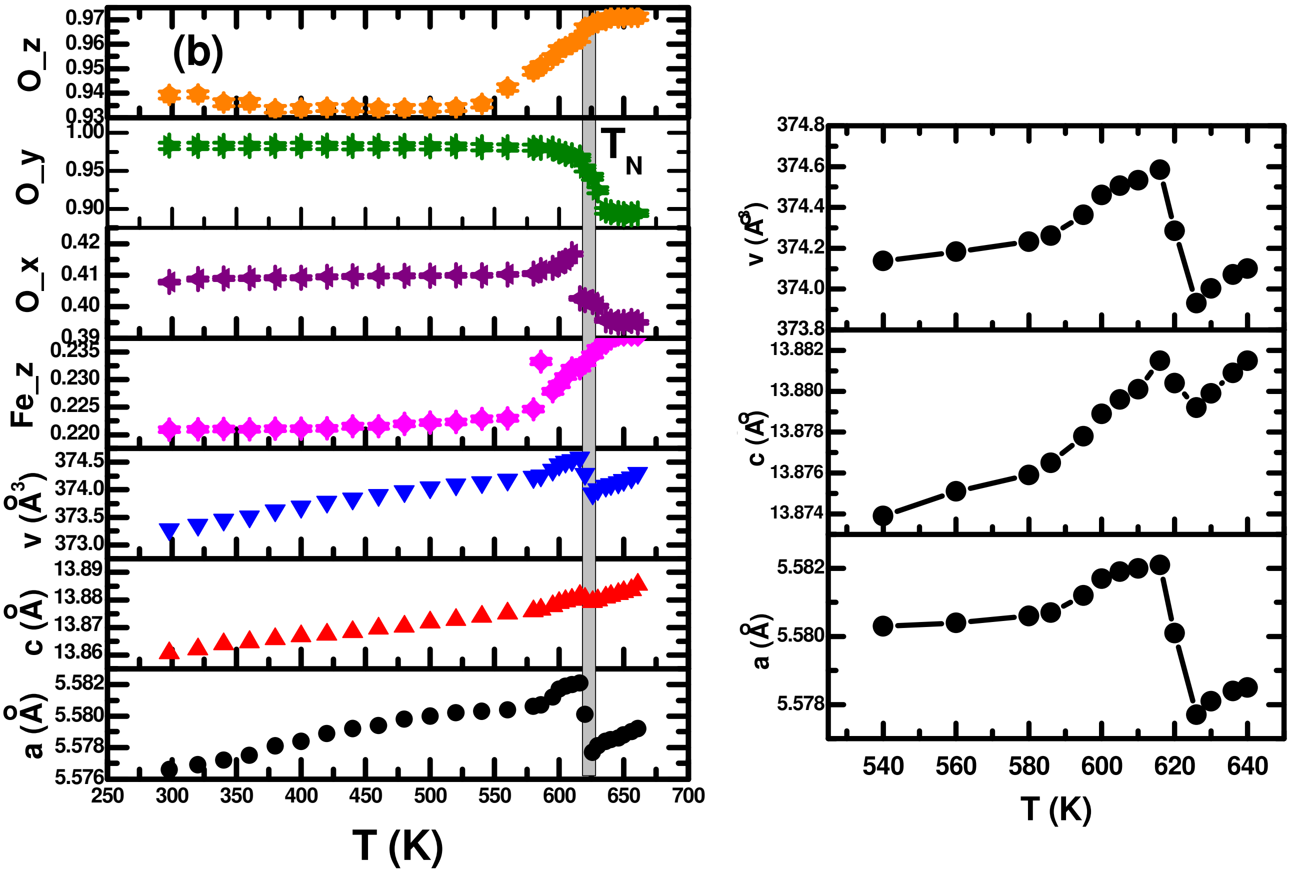
(b)
O_z
O_y
O_x
Fe_z
v (Å³)
c (Å)
a (Å)
T_N
T (K)

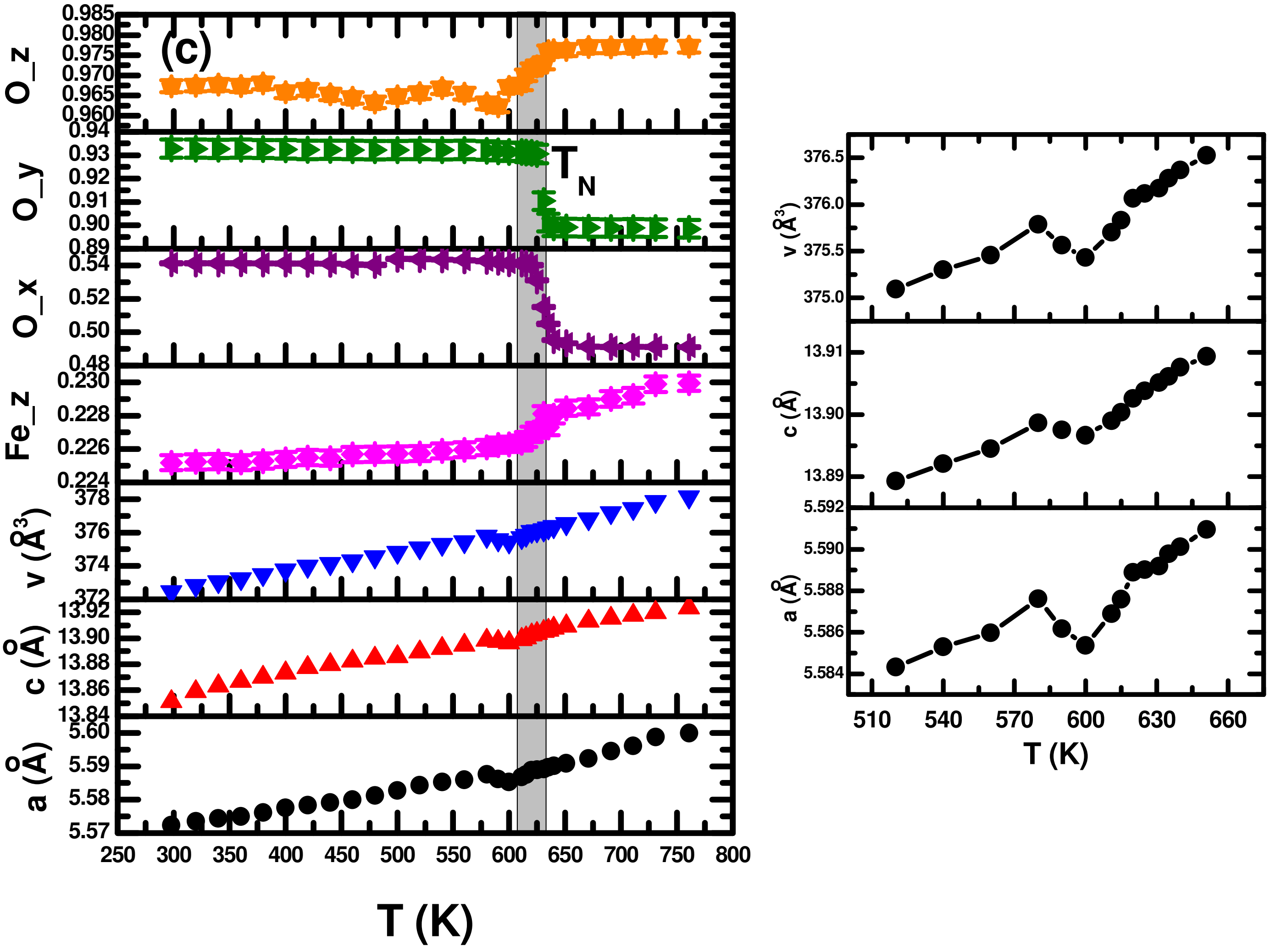

Fig. S8. Temperature dependence of structural parameters for (a) ~30 nm, (b) ~60 nm, and (c) ~100 nm particles of $BiFeO_3$; the region near transition is blown up in the side panels to show the contraction of the lattice parameters and volume around the transition at T_N. The error bars have been drawn by using the estimated standard deviation obtained from Rietveld refinement. For the lattice parameters they vary within 0.2-0.4%.

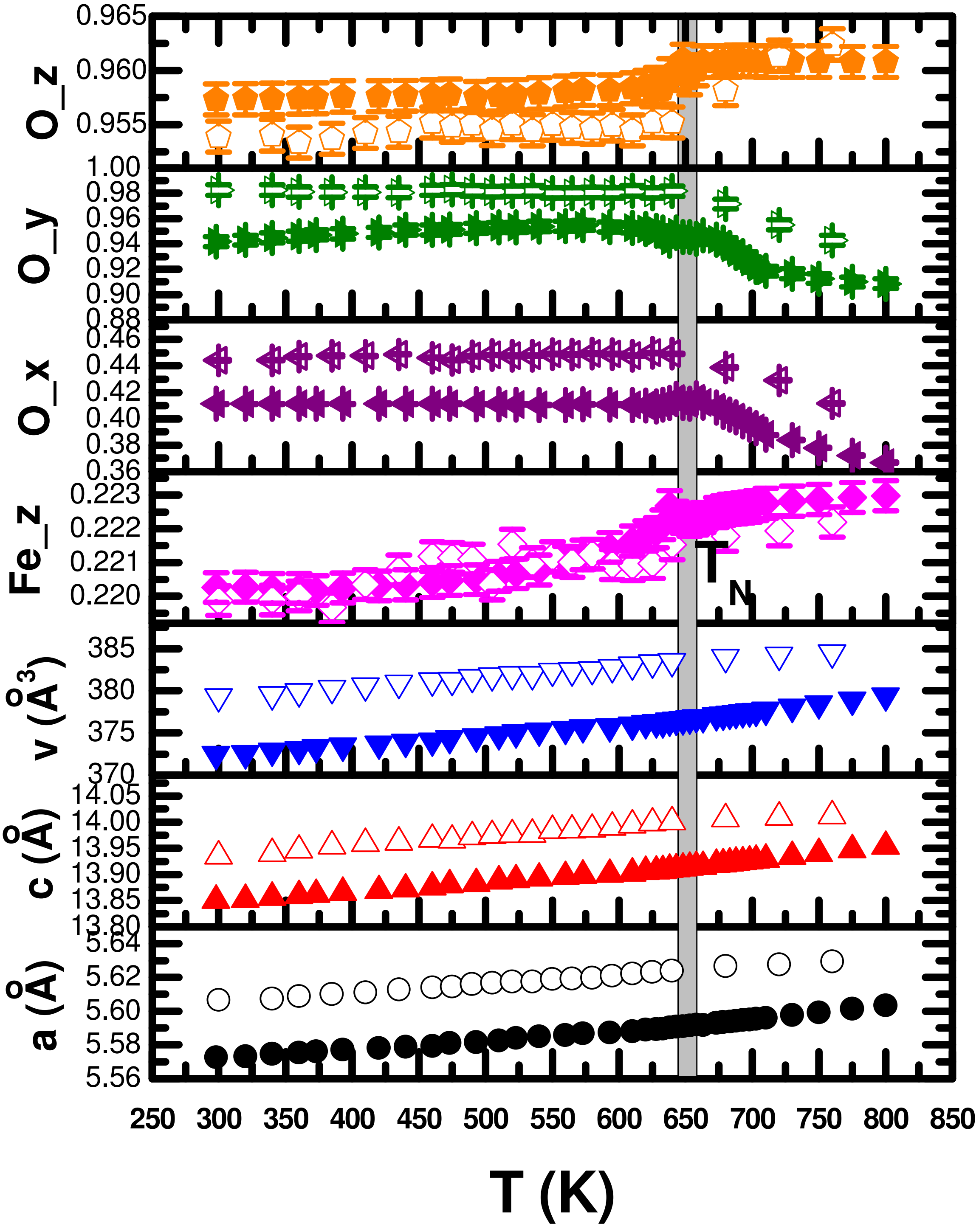

Fig. S9. Comparison between results obtained from x-ray (solid symbols) and neutron diffraction (open symbols) for bulk sample. The error bars are drawn by using standard deviation obtained from Rietveld refinement. In the case of lattice parameters the errors vary within 0.2-0.4%.

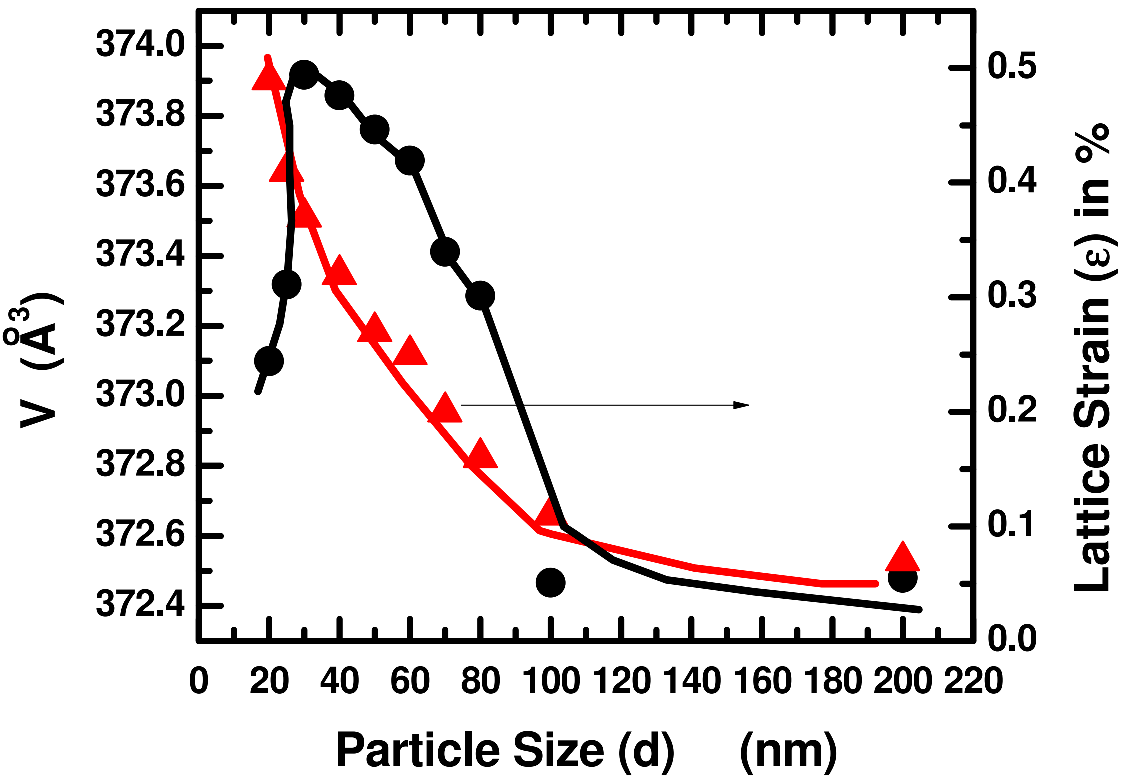

Fig. S10. Particle size dependence of lattice volume and strain.

Table S-II. Ion displacement modes obtained for R3c space group.

For k = 0 structure, the Fe(6a) modes decomposed into following irreducible representations $\tau_{6a} = \tau_1 + \tau_2 + 2\tau_3$; the O(18b) modes can be decomposed into the irreducible representations $\tau_{18b} = 3\tau_1 + 3\tau_2 + 6\tau_3$.

**Irred. Repres.**	**Basis Vectors**		**6a site**		**18b site**							
			**(0,0,z)**	**(0,0,z+½)**	**(x,y,z)**			**(-y,x-y,z)**	**(-x+y,-x,z)**	**(-y,x,z+½)**	**-x+y,y,z+ ½)**	**x,x-y,z+½)**
τ_1	D^1_1		(001)	(001)	D^1_1		(100)	(010)	(-1-10)	(0-10)	(-100)	(110)
					D^1_2		(010)	(-1-10)	(100)	(-100)	(110)	(0-10)
					D^1_3		(001)	(001)	(001)	(001)	(001)	(001)
τ_2	D^2_1		(001)	(00-1)	D^2_1		(100)	(010)	(-1-10)	(010)	(100)	(-1-10)
					D^2_2		(010)	(-1-10)	(100)	(100)	(-1-10)	(010)
					D^2_3		(001)	(001)	(001)	(00-1)	(00-1)	(00-1)
τ_3	D^3_1	Re	(3/200)	(000)	D^3_1	Re	(100)	(0-½ 0)	(½½0)	(000)	(000)	(000)
		Im	(-√3/2-√30)	(000)		Im	(000)	(0-√3/20)	(-√3/2-√3/20)	(000)	(000)	(000)
	D^3_2	Re	(000)	(0-1.50)	D^3_2	Re	(010)	(1/21/20)	(-½00)	(000)	(000)	(000)
		Im	(000)	(-√3-√3/20)		Im	(000)	(√3/2√3/20)	(√3/200)	(000)	(000)	(000)
	D^3_3	Re	(000)	(0-1.50)	D^3_3	Re	(001)	(00-½)	(00-½)	(000)	(000)	(000)
		Im	(000)	(√3√3/20)		Im	(000)	(00-√3/2)	(00√3/2)	(000)	(000)	(000)
	D^3_4	Re	(1.500)	(000)	D^3_4	Re	(000)	(000)	(000)	(0-10)	(½00)	(-½-½0)
		Im	(√3/2√30)	(000)		Im	(000)	(000)	(000)	(000)	(-√3/200)	(-√3/2-√3/20)
					D^3_5	Re	(000)	(000)	(000)	(-100)	(-½ -½0)	(0½0)
						Im	(000)	(000)	(000)	(000)	(√3/2√3/20)	(0√3/20)
					D^3_6	Re	(000)	(000)	(000)	(001)	(00-½)	(00-½)
						Im	(000)	(000)	(000)	(000)	(00√3/2)	(00-√3/2)
					D^3_7	Re	(000)	(000)	(000)	(0-10)	(½00)	(-½-½0)
						Im	(000)	(000)	(000)	(000)	(√3/200)	(√3/2√3/20)
					D^3_8	Re	(000)	(000)	(000)	(-100)	(-½-½0)	(0½0)
						Im	(000)	(000)	(000)	(000)	(-√3/2-√3/20)	(0-√3/20)
					D^3_9	Re	(000)	(000)	(000)	(001)	(00-½)	(00-½)
						Im	(000)	(000)	(000)	(000)	(00-√3/2)	(00√3/2)
					D^3_{10}	Re	(100)	(0-½0)	(½½0)	(000)	(000)	(000)
						Im	(000)	(0√3/20)	(√3/2√3/20)	(000)	(000)	(000)
					D^3_{11}	Re	(010)	(½½0)	(-½00)	(000)	(000)	(000)
						Im	(000)	(-√3/2-√3/20)	(-√3/200)	(000)	(000)	(000)
					D^3_{12}	Re	(001)	(00-½)	(00-½)	(000)	(000)	(000)
						Im	(000)	(00√3/2)	(00-√3/2)	(000)	(000)	(000)

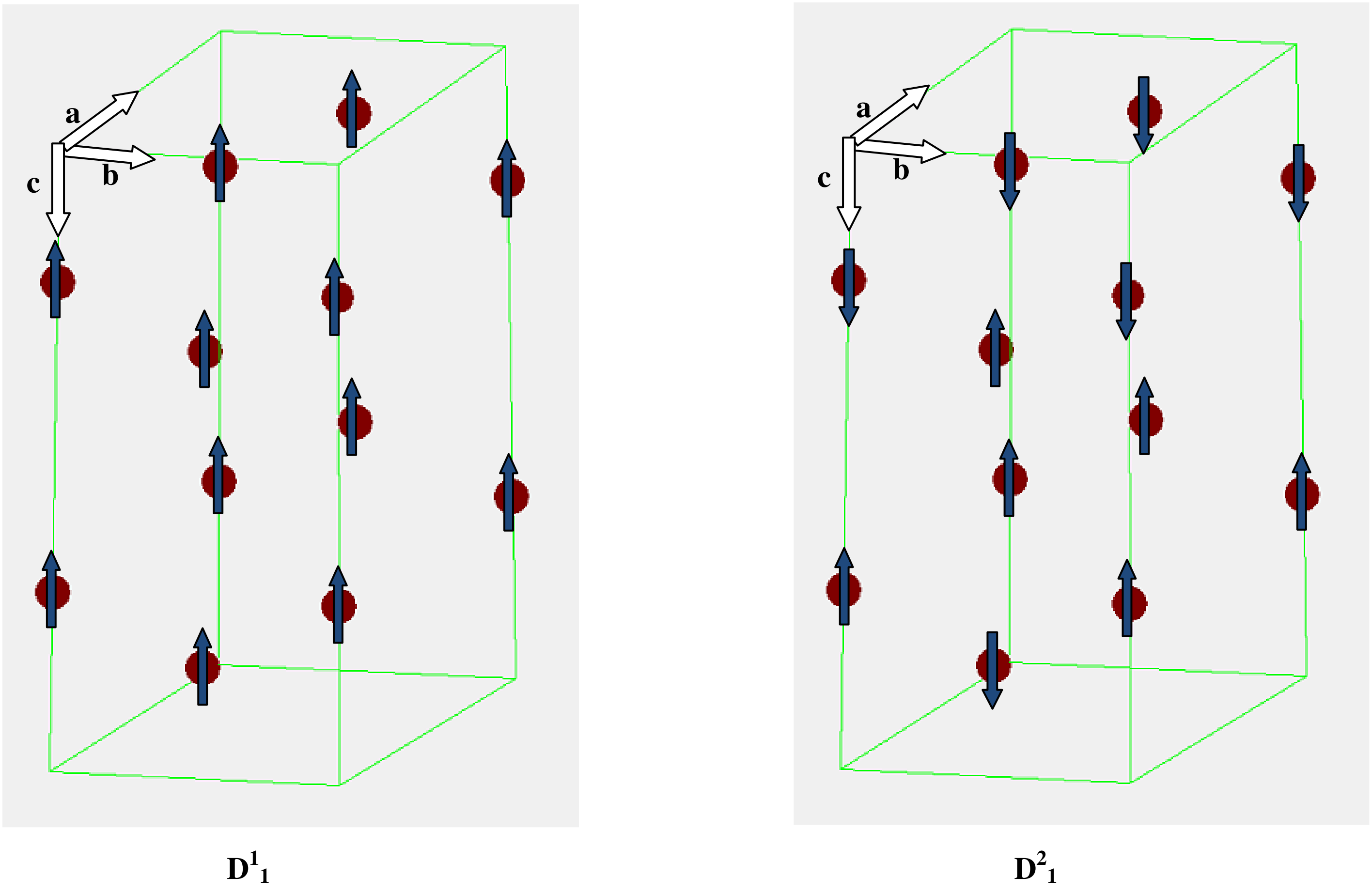

Fig. S11. Displacement modes for Fe (6a) ions for τ_1 ($D^1{}_1$) and τ_2 ($D^2{}_1$).

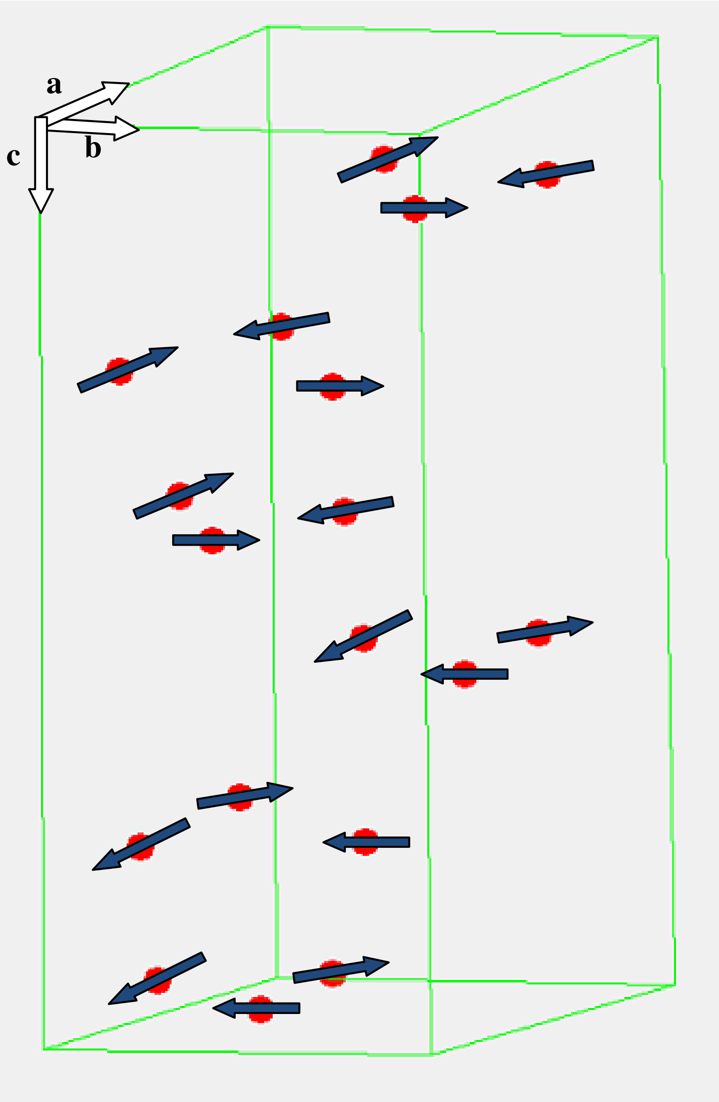

D^1_1

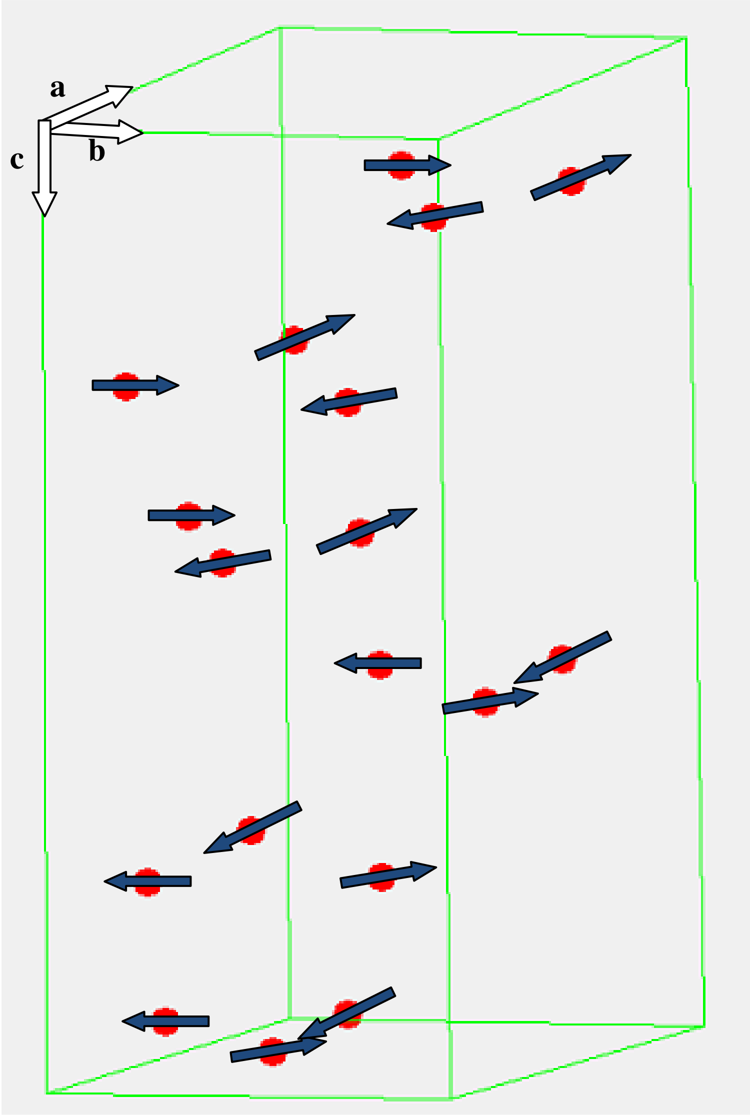

D^1_2

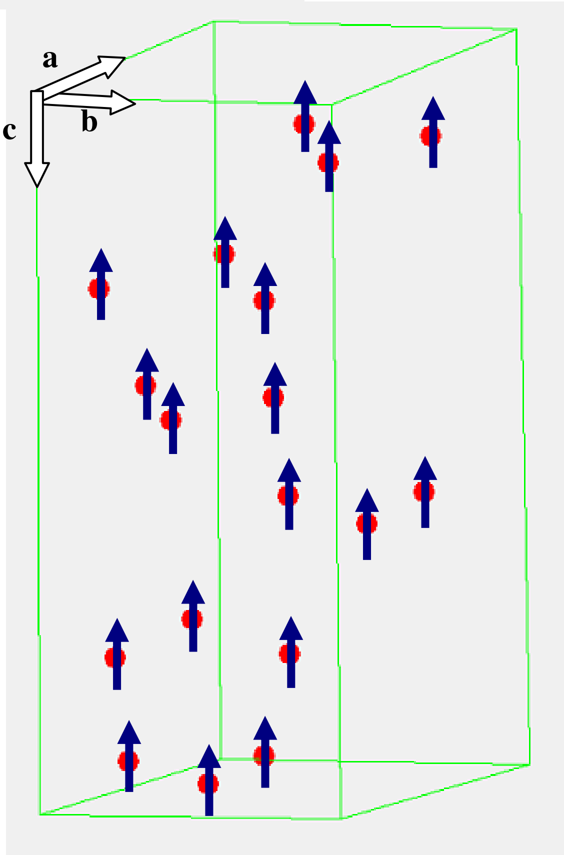

D^1_3

Fig. S12. Displacement modes for O (18b) ions for τ_1.

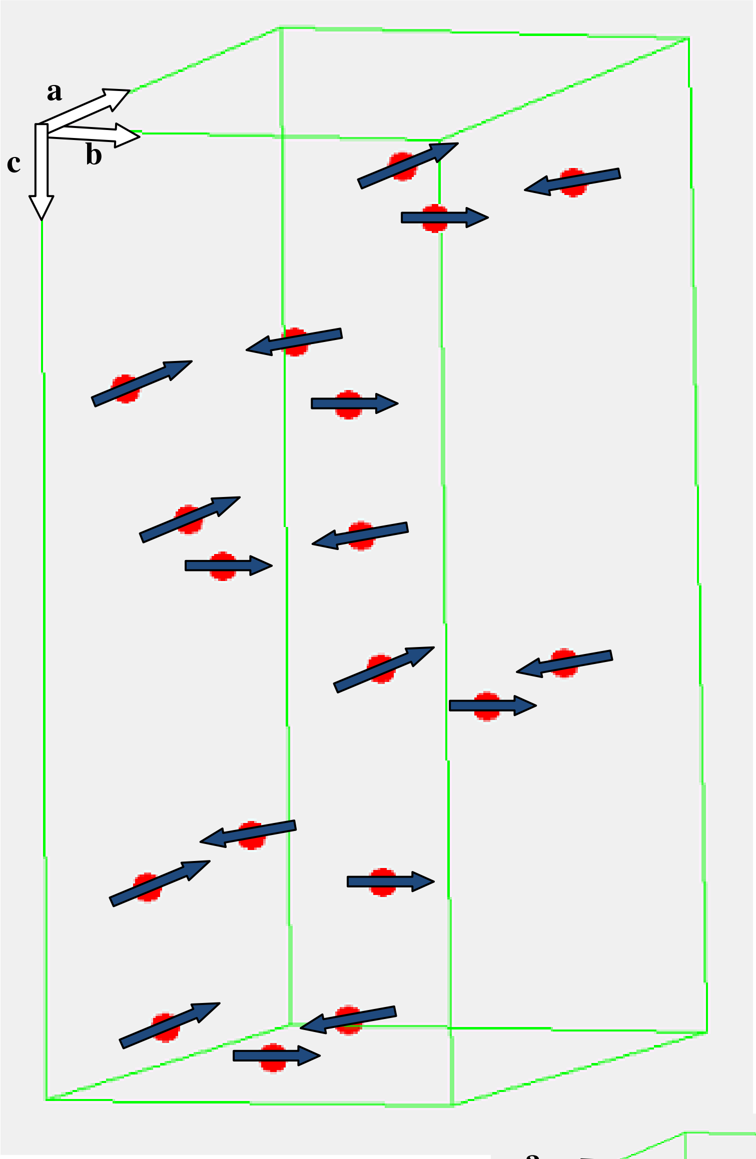

$D^2{}_1$

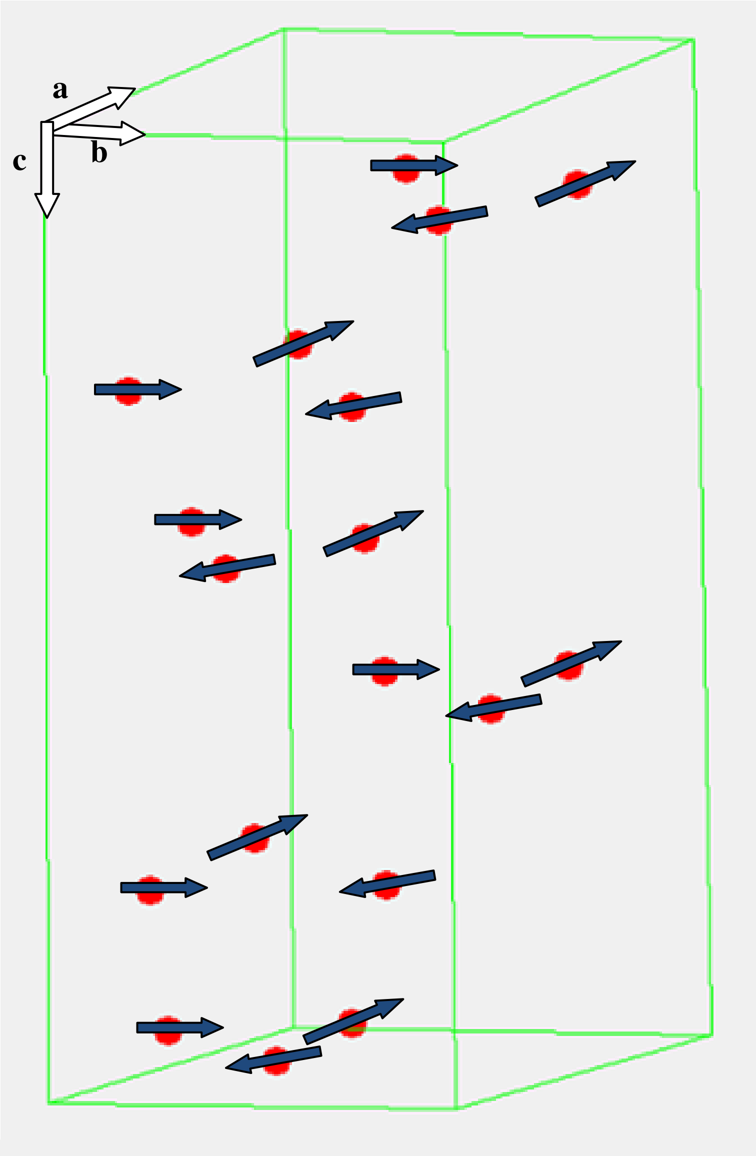

$D^2{}_2$

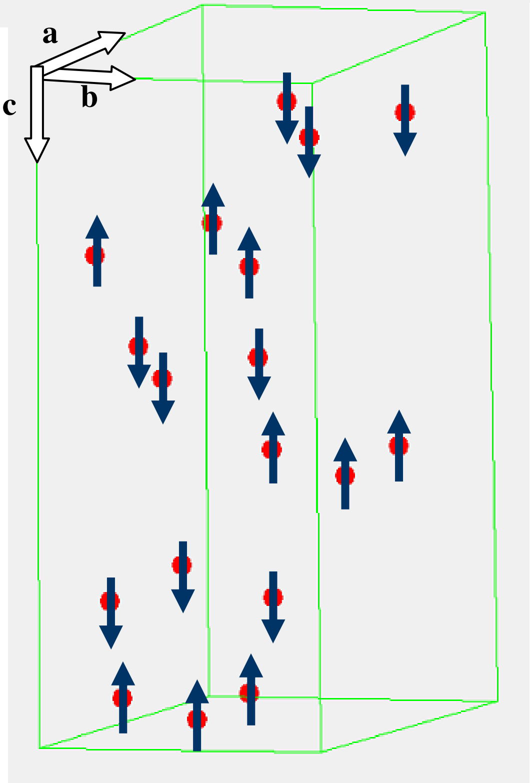

$D^2{}_3$

Fig. S13. Displacement modes for O (18b) ions for τ_2.

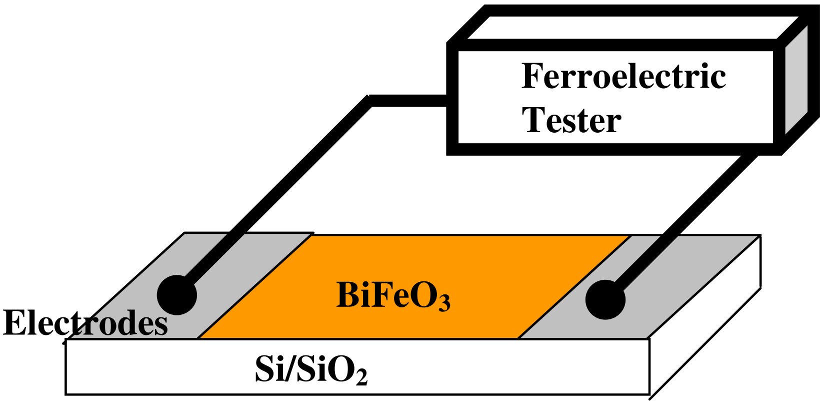

Fig. S14. The schematic of the sample-electrode configuration used for measurement of the remanent ferroelectric hysteresis loops under zero and ~20 kOe magnetic field.
